# Evaluation of Dosimetry Check software for IMRT patient‐specific quality assurance

**DOI:** 10.1120/jacmp.v16i3.5427

**Published:** 2015-05-08

**Authors:** Ganesh Narayanasamy, Travis Zalman, Chul S. Ha, Niko Papanikolaou, Sotirios Stathakis

**Affiliations:** ^1^ Department of Radiation Oncology University of Texas Health Science Center at San Antonio TX USA

**Keywords:** dosimetry check, IMRT QA, QA techniques, dosimetry

## Abstract

The purpose of this study is to evaluate the use of the Dosimetry Check system for patient‐specific IMRT QA. Typical QA methods measure the dose in an array dosimeter surrounded by homogenous medium for which the treatment plan has been recomputed. With the Dosimetry Check system, fluence measurements acquired on a portal dosimeter is applied to the patient's CT scans. Instead of making dose comparisons in a plane, Dosimetry Check system produces isodose lines and dose‐volume histograms based on the planning CT images. By exporting the dose distribution from the treatment planning system into the Dosimetry Check system, one is able to make a direct comparison between the calculated dose and the planned dose. The versatility of the software is evaluated with respect to the two IMRT techniques — step and shoot and volumetric arc therapy. The system analyzed measurements made using EPID, PTW seven29, and IBA MatriXX, and an intercomparison study was performed. Plans from patients previously treated at our institution with treated anatomical site on brain, head & neck, liver, lung, and prostate were analyzed using Dosimetry Check system for any anatomical site dependence. We have recommendations and possible precautions that may be necessary to ensure proper QA with the Dosimetry Check system.

PACS number: 87.55.Qr, 87.56.Fc

## I. INTRODUCTION

An early technique used for patient‐specific quality assurance (QA) was combining an ionization chamber and film. Ion chambers provide excellent stability, linear response to absorbed dose, small directional dependence, beam‐quality response independence, and traceability to a primary calibration standard.^1^, ^2^ Films are convenient to use, provide excellent spatial resolution, and acts as a permanent record of the integrated planar dose distributions.^(3)^ By using a water‐equivalent phantom with the capability of supporting an ion chamber and films, one could verify both absolute and relative dose for an intensity‐modulated radiation therapy (IMRT) treatment with any analyzing software.

Two‐dimensional (2D) arrays provide a quick and efficient means of performing patient‐specific QA by eliminating the need for film processing. These detectors typically employ diode or ion chamber array which are calibrated to provide both absolute and relative dose measurements immediately after irradiation.

Although electronic portal imaging dosimeter (EPID) was designed to provide patient position verification, interest in portal dosimetry is growing due to high spatial resolution, large imaging area, stability, dynamic range, and real‐time acquisition of images.^4^, ^5^, ^6^ Typically, the use of an EPID for patient‐specific QA is limited to 2D dose distributions. The Dosimetry Check system investigated in this study has taken this approach one step further by employing a pencil beam algorithm in order to calculate dose from fluence measurements taken with the EPID.^(7)^ It can be assumed that some dose differences will arise from the pencil beam algorithm used in Dosimetry Check and the more sophisticated algorithms used in TPS, but it is believed that these differences should be no greater than 5%.^(7)^

## II. MATERIALS AND METHODS

In the first part of the study, linearity of Dosimetry Check system with dose delivered is studied using a 10 cm×10 cm 6 MV photon beam in order to ensure that the system operates as desired. All measurements were taken with a 6 MV photon beam produced by a Varian Novalis TX linac (Varian Medical Systems, Palo Alto, CA) equipped with a HD120 MLC system. Pinnacle^3^ TPS (Philips Medical Systems, Andover, MA) used at our institution employs a convolution/superposition algorithm. Frame rate dependence of the system is studied with an integrated image for step‐and‐shoot IMRT plans versus a cine‐mode continuous image for the arc therapy plans. The dependence of the Dosimetry Check system on the detectors was analyzed by comparing EPID based measurements against other QA detectors including PTW seven29 ion chamber array (PTW, Freiburg, Germany) and IBA MatriXX ion chamber array (IBA Dosimetry, Bartlett, TN). The amorphous‐Si (aSi1000) EPID is an array of 1024×768 a‐Si based photodiodes arranged in a $40\times 30\;{\text{cm}}^{2}$ active area. The PTW seven29 array consists of 729 vented ionization chambers (0.125 cc active volume) arranged in a matrix of 27×27 chambers with center‐to‐center spacing of 10 mm.^(8)^ The IBA I'mRT MatriXX array comprises of 1020 vented ion chambers (0.08 cc active volume) arranged in a matrix of 32×32 chambers with center‐to‐center spacing of 7.62 mm.^(9)^

As a part of this study, treatment plans from five different anatomical sites (brain, head and neck, liver, lung, and prostate) were chosen to check for any anatomical site dependence of the EPID‐based Dosimetry Check analysis. For dosimetric verification, gamma analysis was performed using 3% dose difference, 3 mm distance‐to‐agreement criteria, with a 5% dose threshold upon normalizing to the prescription dose.

### A. Verification of linearity

Linearity of the QA dosimetric system allows for the usage of a single integrated image to calibrate all fields. To verify the linearity of the detectors, five fields of size 10 cm×10 cm with increasing MUs are irradiated on the EPID, seven29, and MatriXX. For the purposes of this study, both the seven29 and MatriXX were positioned on the couch at a source‐to‐detector distance (SDD) of 100 cm. However, EPID dosimeter was set at the highest possible setting of 110 cm SDD, allowing us to protect the electronics of EPID. This necessitates the Dosimetry Check software to make the distance corrections for differences in SDD. The integrated images were then imported into the Dosimetry Check system and the signal values for each image were plotted against the MUs to establish the calibration file.

### B. Software evaluation

The Dosimetry Check software was used to evaluate patient‐specific QA on the two IMRT techniques — step and shoot, as well as smart arc with measurements based on EPID set to 110 cm SDD. With step‐and‐shoot IMRT plans, it is only necessary to have one integrated image per field. In a smart arc plan, a continuous image technique allows the EPID to collect multiple integrated images at a frame averaging selected by the user. A balance between the timer resolution and the number of images was achieved using a frame averaging that gives an image within every five degrees of gantry rotation. Frame averaging is selected according to the gantry rotation speed and the degree of modulation. It is possible that a loss of signal may occur in the duration between two consecutive image acquisitions and, therefore, an integrated image of each arc is also taken. The Dosimetry Check system applies a correction factor of the ratio of the sum of the signal from the continuous images (acquired at every 50 gantry rotation) and the single integrated image (acquired for the whole arc) during dose reconstruction. Gamma analysis was then used to compare the measured data with the reference data using a 3%/3 mm criteria and a 5% dose threshold after normalizing to the prescription dose.

**Figure 1:**
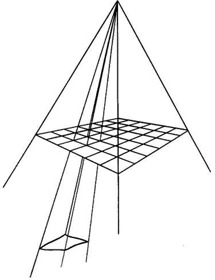
Back‐projection and pencil beam calculation.

**Figure 2:**
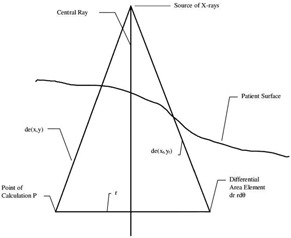
Pencil beam algorithm used in dosimetry check.

The Dosimetry Check system uses integrated images of radiation fields in order to calculate dose intended to be delivered. The X‐ray intensity map measured with EPID is converted to relative monitor units by using a calibration file. The calibration files are created by taking the central axis value of a $10\times 10\;{\text{cm}}^{2}$ image with a known amount of monitor units (MU). This process is repeated with increasing MUs to study the linearity of Dosimetry Check system.^(10)^ Once the integrated images are calibrated, they are transformed into incident intensity fluence by spatial filtering with a deconvolution kernel.^(11)^ The fluence fields can then be back‐projected (shown in Fig. 1) and the dose is calculated using a pencil beam algorithm (shown in Fig. 2). The pencil beam was developed from the commissioning data and the Monte Carlo calculated polyenergetic kernel.^(7)^ After importing the CT scans, plan file, structure file, and dose file from the treatment planning software (TPS), a direct comparison between computed dose and planned dose can be made not only in a plane but in the entire body or specific organs of the patient. The Dosimetry Check system also provides the user with isodose lines, point doses, dose‐volume histograms (DVH), and gamma analysis in order to make these direct comparisons.

### C. Detector dependence

**Figure 3:**
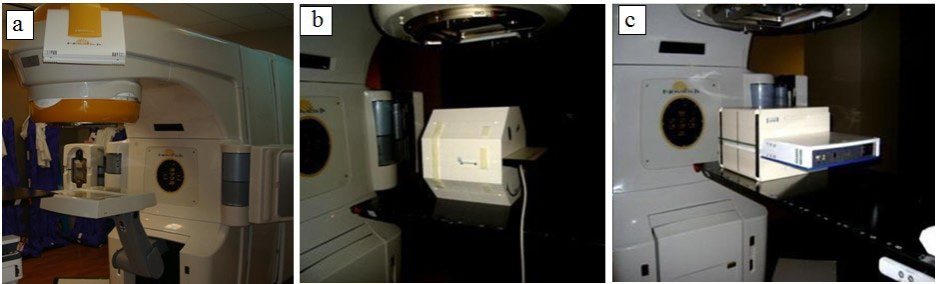
Picture of the setup: (a) EPID, (b) PTW seven29 array, and (c) IBA I'mRT MatriXX.

**Figure 4:**
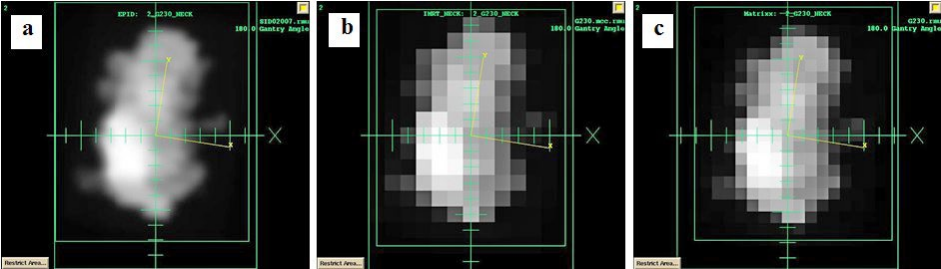
Calibrated fluence fields for (a) EPID, (b) PTW seven29, and (c) IBA I'mRT MatriXX.

**Figure 5:**
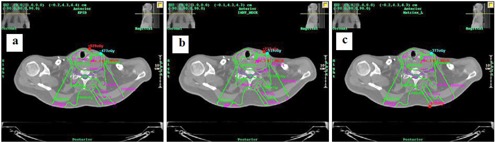
Isodose lines in the reference data from TPS (green) and calculated with Dosimetry Check system (magenta) on the same patient with (a) aSi1000 EPID, (b) PTW seven29, and (c) IBA I'mRT MatriXX.

Fifteen step‐and‐shoot IMRT plans from various treated anatomical sites were chosen from patients previously treated at our institution. Integrated images were taken for each field for the 15 plans with all three detectors, with setup as shown in Fig. 3. Each field was delivered with the gantry set to upright position and SDD of 110 cm for the EPID and 100 cm for both the PTW seven29 and IBA I'mRT Matrixx. A 10 cm×10 cm field with 100 MUs was delivered for calibration to account for the daily variations of the linac. The images were then imported into the Dosimetry Check system along with the CT scans, structure file, plan file, and dose distribution file exported from the TPS. The Dosimetry Check system uses the imported fluence fields (shown in an example in Fig. 4) to calculate the absolute dose, and isodose curves (example shown in Fig. 5) to compare with the reference data. Gamma analysis was used to compare the measured data with the reference data using the above‐mentioned criteria. Bland‐Altman test is performed in analyzing the agreement between two methods of measurement. In this study, pairwise comparison between EPID and seven29, EPID and MatriXX, and seven29 and MatriXX were performed using Bland‐Altman tests to identify the agreement and the presence of any bias.

### D. Anatomical site dependence

The dose algorithm used in the Dosimetry Check system is a pencil beam algorithm which differs from the convolution/superposition algorithm widely used in the TPSs. Differences in these algorithms may lead to discrepancies in the dose calculated with the Dosimetry Check system and may depend on the level of heterogeneity of the anatomical site. In the anatomical site‐dependence study, EPID‐based measurements were collected on 23 step‐and‐shoot IMRT plans from five different anatomical sites (brain, head & neck, liver, lung, and prostate). Dose calculated by Dosimetry Check system was compared with the TPS reference dose using 3D gamma analysis.

## III. RESULTS

**Figure 6:**
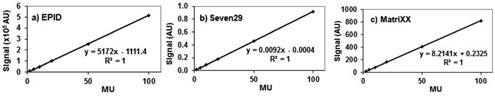
Output signal linearity plots against MUs in a 10 cm×10 cm 6 MV open beam for (a) EPID, (b) seven29, and (c) MatriXX.

In the linearity study, five measurements were taken at each MU setting to verify the consistency in the measurements. The mean values were plotted and a linear fit with the regression calculated was applied to each set of data, as shown in Fig. 6. The R^2^ value for each plot was 1, clearly showing the dose response of each detector is linear. The standard deviation (SD) in the measurements is `1%, except for seven29 and MatriXX where it is 3.7% and 1.3% at 2MUs, respectively. Mean percent SD values decrease from 1.74% to 0.05% with increasing MUs, showing more consistency across the three detectors.

In evaluating the Dosimetry Check software on step‐and‐shoot IMRT plans and arc therapy plans, ten plans of each type from patients previously treated at our institution were measured. While integrated images were acquired in the step‐and‐shoot IMRT plans, continuous images were measured in the arc therapy plans treatment type. Gamma analysis between the measured and the reference plan dose yielded mean ±SD of 96%±1% for the step‐and‐shoot IMRT plans. The agreement is slightly higher at 98.9%±0.6% for the arc therapy plans, although no statistically significant difference was observed between the two treatment techniques using a two‐tailed Student's *t*‐test (p>0.1).

**Figure 7:**
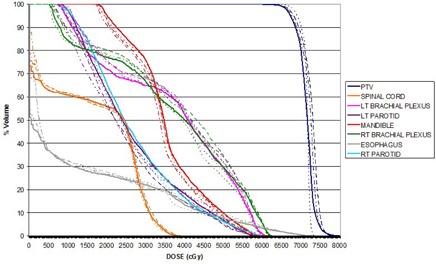
Dosimetry Check system calculated DVH comparing the aSi1000 EPID (solid), PTW seven29 (dashed), IBA I'mRT Matrixx (dashed/dotted), with reference DVH from TPS (dotted) for a head/neck plan.

**Figure 8:**
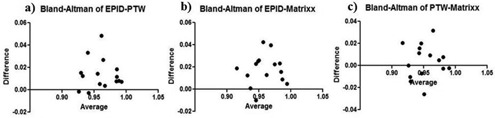
Results from Bland‐Altman test analysis comparing gamma values between (a) EPID–PTW seven29, (b) EPID–MatriXX, and (c) PTW seven29–MatriXX.

In the detector dependency study, integrated images were taken for each field of the 15 step‐and‐shoot IMRT plans with all three detectors. These plans correspond to various anatomical sites from patients treated earlier at our institution. The measured detector fluence data and the patient CT scans were imported into the Dosimetry Check system to calculate DVH of various organs. Figure 7 shows the DVH calculated by Dosimetry Check system and the reference DVH from the TPS for a head/neck IMRT plan. Gamma analysis was used to compare the measured and reference data using 3%/3 mm criteria and a 5% dose threshold was used after normalizing to the prescription dose. The gamma values for the 15 plans are listed, along with the mean and SD value, in Table 1. Three Bland‐Altman tests were performed in order to compare the measured data and results are shown in Fig. 8. Each Bland‐Altman test performed a pairwise comparison between the gamma values calculated using imported fluence from two of the detectors. The bias ±SD values were 0.01391±0.01357 and 0.01775±0.01379 for comparisons between the EPID and seven29 and MatriXX, respectively. The bias value for the comparison between the 2D arrays was 0.003847±0.01515.

**Table 1:** Gamma values calculated by Dosimetry Check system with fluence from EPID, seven29, and MatriXX on 15 IMRT plans.

*Anatomical Site*	*EPID*	*seven29*	*Matrixx*
Abdomen	98.85%	98.08%	94.90%
Brain 1	97.77%	95.09%	93.51%
Brain 2	94.03%	94.30%	95.03%
Brain 3	99.26%	98.45%	97.70%
Head and Neck 1	92.51%	92.68%	90.62%
Head and Neck 2	98.59%	93.74%	96.33%
Head and Neck 3	96.86%	96.48%	95.56%
Liver 1	99.09%	97.93%	98.22%
Liver 2	99.62%	98.90%	99.13%
Liver 3	99.46%	97.61%	97.13%
Lung 1	93.71%	92.19%	93.64%
Lung 2	95.66%	92.34%	93.38%
Lung 3	96.12%	95.59%	93.59%
Prostate 1	93.85%	92.60%	92.61%
Prostate 2	96.28%	94.82%	93.68%
Average	96.78%	95.39%	95.00%
SD	2.41%	2.40%	2.34%

**Figure 9:**
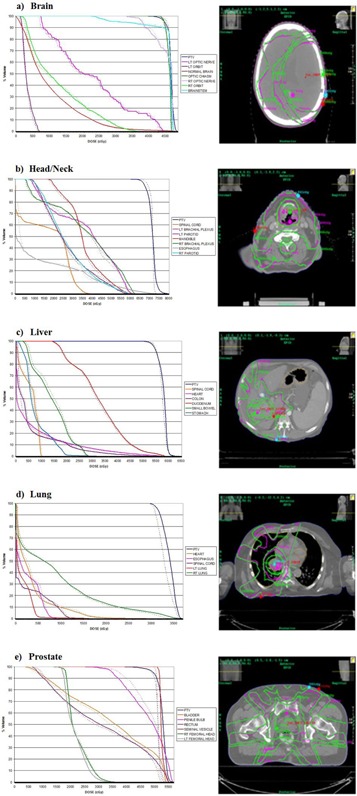
DVH and isodose lines in the reference data from TPS (green) can be compared with those calculated by the Dosimetry Check system (magenta) for: (a) brain, (b) head and neck, (c) liver, (d) lung, and (e) prostate treatment plans.

**Figure 10:**
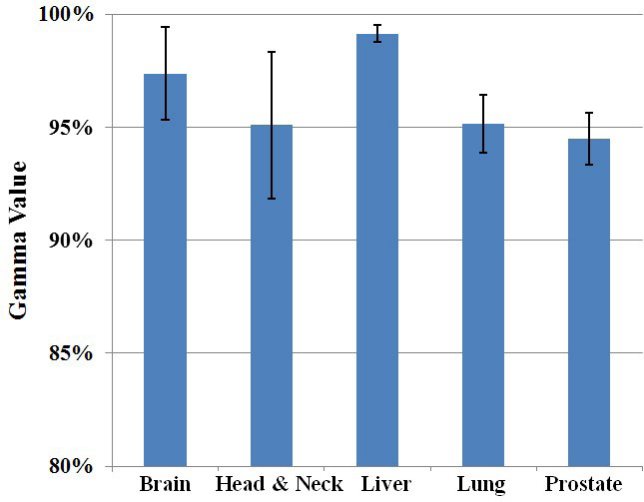
Breakdown of gamma analysis values from the EPID–Dosimetry Check system calculated data according to the anatomical site on the 23 IMRT plans. Error bars represent 1 SD.

In the anatomical site dependency study, 23 IMRT plans were chosen from patients previously treated at our institution. The Dosimetry Check system calibrated the imported fluence fields collected as integrated images on EPID along with the reference data from the TPS to calculate the absolute dose, isodose curves, and DVHs. Shown in Fig. 9 are the reference and calculated isodose curves and DVHs for five treatment plans — brain, head/neck, liver, lung and prostate. The gamma values for the 23 plans are listed in Table 2 and the average SD values are plotted according to anatomical site in Fig. 10. Small anatomical site dependence was observed. Of the sites investigated, liver and brain patients showed the strongest agreement between calculated and reference data, with mean gamma values of 99.15% and 97.38%, respectively. The remaining sites had mean gamma values between 94.49% and 95.16%. Of note is the lung plan where an over estimation of dose to the PTV in the lung is observed in the pencil beam algorithm, as shown in Fig. 9(d).

**Table 2:** Dosimetry Check system calculated gamma values on 23 IMRT patient plans to various anatomical sites.

*Anatomical Site*	*Gamma*
Brain 1	97.77%
Brain 2	94.03%
Brain 3	99.26%
Brain 4	97.10%
Brain 5	98.72%
Head and Neck 1	96.41%
Head and Neck 2	98.59%
Head and Neck 3	92.99%
Head and Neck 4	90.59%
Head and Neck 5	96.86%
Liver 1	98.75%
Liver 2	99.09%
Liver 3	98.81%
Liver 4	99.62%
Liver 5	99.46%
Lung 1	93.71%
Lung 2	95.66%
Lung 3	96.12%
Prostate 1	93.85%
Prostate 2	96.28%
Prostate 3	93.79%
Prostate 4	94.91%
Prostate 5	93.60%

## IV. DISCUSSION

The first aspect of the software we investigated was the ability to perform patient‐specific quality assurance with different treatment techniques. The linear dose response characteristic of each detector allowed us to take a single image for calibration for each patient QA. A calibration image was taken on the day of making patient‐specific QA measurements instead of using a calibration file, in order to take into account the daily output variation of the linac.

In our institution we currently employ both step‐and‐shoot treatments, as well as smart arc treatments, and the Dosimetry Check software was able to perform adequately for both treatments. The difference between testing these two treatment techniques lies in the image acquisition mode. A slightly higher gamma value is observed in the arc plans, although the difference is negligible.

The next aspect that was investigated is the ability of the system to use fluence maps from various detectors for patient‐specific QA. The Dosimetry Check system is not restricted to work only with EPID, but allows fluence maps from a number of different detectors. A group of 15 step‐and‐shoot IMRT patient plans were chosen from various anatomical sites and fluence maps were taken with three separate detectors, the aSi1000 EPID, PTW seven29, and IBA I'mRT Matrixx. Gamma analysis and Bland‐Altman tests showed that all three detectors performed adequately, with negligible differences.

In the anatomical site dependence test, 23 IMRT plans from five anatomical sites with varying levels of inhomogeneity were investigated. Inherent differences between a pencil beam algorithm employed in Dosimetry Check system and the superposition/convolution algorithm used in Pinnacle^3^ TPS can result in dose discrepancies between the calculated and reference dose. The pencil beam algorithm used in the Dosimetry Check system is not expected to compute dose accurately at the internal interfaces, especially when compared with a convolution/superposition algorithm.^(7)^ Pencil beam algorithms are known to overestimate dose in low density inhomogeneity areas.^(12)^ In one of the lung plans shown in Fig. 9, the dose calculated for the PTV is higher using a pencil beam algorithm compared to the reference DVH. While some anatomical site dependence does occur, our investigation showed that the differences are small and the Dosimetry Check system was able to perform well in QA for all anatomical sites. Caution is recommended with the use of this system when inhomogeneity exists, and it is recommended to take a close look at the DVH and isodose lines.

## V. CONCLUSIONS

Dosimetry Check system is a very versatile software in its ability to perform patient‐specific QA for various treatment techniques, anatomical sites, and detectors that can be used. Most other QA techniques can only perform a gamma analysis in a single plane. By employing its own dose calculation algorithm, the dose can be calculated in three dimensions, and the isodose lines and DVH can be generated for comparison against the reference data. Another advantage in employing a pencil beam algorithm is the ability to calculate dose in less time. An arc treatment has over 100 images and dose calculation time ranging from a few seconds up to a minute per image, could become a concern. Overall, the Dosimetry Check system provides a versatility and wealth of knowledge that can provide a strong base for a clinical patient‐specific QA program.

## Supporting information

Supplementary Material

Supplementary Material
