# Supplementary material for: Evaluation of Dosimetry Check software for IMRT patient‐specific quality assurance
Source: J Appl Clin Med Phys. 2015 May 8;16(3):329–38. doi: 10.1120/jacmp.v16i3.5427 (PMC5690116; doi:10.1120/jacmp.v16i3.5427)
Supplement: Supplementary file 1 — Supplementary Material [file ACM2-16-329-s001.docx]

Evaluation of dosimetry check software for imrt patient specific quality assurance

Ganesh Narayanasamy, Travis Zalman, Chul S. Ha, Niko Papanikolaou, Sotirios Stathakis,

Department of Radiation Oncology, University of Texas Health Science Center at San Antonio

Running Title: Evaluation of Dosimetry Check Software for IMRT QA

Keywords: Dosimetry check, IMRT QA, QA technique, Dosimetry
